# Body mass index and dietary intake as nutritional determinants of sarcopenia in older adults

**DOI:** 10.3389/fnut.2026.1798406

**Published:** 2026-05-04

**Authors:** De-En Wang, Wei Yang, Xiaofang Qin

**Affiliations:** 1 Geriatric Medicine Department, The Affiliated Huaian No.1 People’s Hospital of Nanjing Medical University, Huaian, China; 2 Ultrasound Medicine Department, The Affiliated Huaian No.1 People’s Hospital of Nanjing Medical University, Huaian, China

**Keywords:** aging, diet quality, energy intake, muscle health, nutritional status, protein intake

## Abstract

**Background:**

Sarcopenia is a progressive, age-associated condition marked by loss of muscle mass, strength, and physical performance, representing a key nutritional and functional problem in aging people. This study examined the relationship of body mass index (BMI) and regular dietary consumption with sarcopenia among older individuals.

**Methods:**

This retrospective analysis evaluated routine geriatric assessment data from adults aged ≥60 years obtained between 2021 and 2023. Sarcopenia was determined utilizing international criteria based on appendicular skeletal muscle mass index, handgrip strength, and gait speed. Regular energy, protein (absolute and relative), and fiber ingestion were examined, and relations were analyzed using multivariable logistic regression adjusted for age, sex, education, physical activity, and hypertension.

**Results:**

Among 360 individuals, 11.4% were identified as sarcopenic. Those with sarcopenia were older in age, less physically active, and had lesser educational attainment. Sarcopenia was associated with lower BMI, decreased muscle mass, poor handgrip strength, and slower gait speed (all *p* < 0.001). Regular energy, protein, and fiber consumptions were lower in sarcopenic subjects. In fully adjusted analysis, decreased BMI (OR = 0.64), reduced protein intake (OR = 0.85), decreased fiber intake (OR = 0.70), and lower energy intake (per 100 kcal/day) (OR = 0.67) were independently associated with increased odds of sarcopenia.

**Conclusion:**

Sarcopenia in older individuals is independently associated with lower BMI and insufficient habitual ingestion of energy, protein, and dietary fiber, underscoring the significant associations with overall nutritional status.

## 1 Introduction

Sarcopenia is an age-related musculoskeletal disease typified by the loss of skeletal muscle mass, muscle strength, and physical performance. It is acknowledged as a significant public health concern in aging cohorts globally due to its relation with decreased mobility, elevated risk of falls, functional impairment, and higher mortality (1). According to recognized worldwide criteria, the prevalence of sarcopenia in persons aged 60–70 years ranges from roughly 5–13% and rises significantly in older age populations (≥80 years) to between 11 and 50% in some cases (2). This variation emphasizes how nutritional, lifestyle, and demographic variables affect the prevalence of sarcopenia.

Nutritional status has frequently been determined as a major factor linked with sarcopenia in older groups. Among regularly used markers, body mass index (BMI), despite its poor ability to differentiate between fat mass and lean mass, remains widely employed in epidemiological and clinical studies as a proxy of overall nutritional status. Multiple observational investigations have shown that decreased BMI is more commonly noticed in older individuals with sarcopenia compared with those without sarcopenia, indicating a relationship between undernutrition and age-associated muscle loss (3, 4). Even though BMI does not directly represent muscle quantity or quality, population-based studies continue to report its usefulness in determining individuals at increased risk of sarcopenia, especially in settings where advanced body composition strategies are not regularly available.

Dietary consumption has also been comprehensively evaluated in relation to sarcopenia and its defining characteristics, such as muscle mass, strength, and physical performance. Protein intake has drawn special at attention due to its crucial role in supplying essential amino acids required for muscle protein synthesis. Evidence from meta-analyses and systematic reviews shows that older persons with sarcopenia consume much less protein in their diets than people without the condition, which supports a link between protein deficiency and poor muscle maintenance as people age (5, 6). The significance of appropriate dietary protein in this population is further supported by observational studies that relate low protein consumption to decreased muscle mass and strength in community-dwelling older persons.

Alongside protein, total energy consumption has been examined as a predictor of sarcopenia. Multiple cross-sectional and cohort investigations indicate that older individuals with sarcopenia have decreased daily energy ingestion than their non-sarcopenic counterparts, reporting that generalized dietary inadequacy may coexist with muscle loss (7, 8). Inadequate calorie intake may worsen negative protein balance, thereby contributing to gradual deterioration in skeletal muscle. Additional nutritional parameters linked to sarcopenia have also been identified, including dietary fiber intake and overall diet quality. After controlling for sociodemographic traits, physical activity, and comorbidities, a number of population-based research have found inverse relationships between dietary fiber consumption and the prevalence of sarcopenia (9–11).

Despite increasing knowledge of nutritional determinants, data evaluation the combined effects of BMI and habitual dietary intake in sarcopenia is still scarce, especially in routine clinical cohorts. Understanding how anthropometric state and major dietary components simultaneously relate to sarcopenia may aid guide targeted nutritional recommendations for older persons. Therefore, this retrospective investigation aimed to evaluate the relationships between BMI, energy intake, protein intake, dietary fiber intake, and sarcopenia among older individuals.

## 2 Methods

### 2.1 Study design and participants

**Figure 1:**
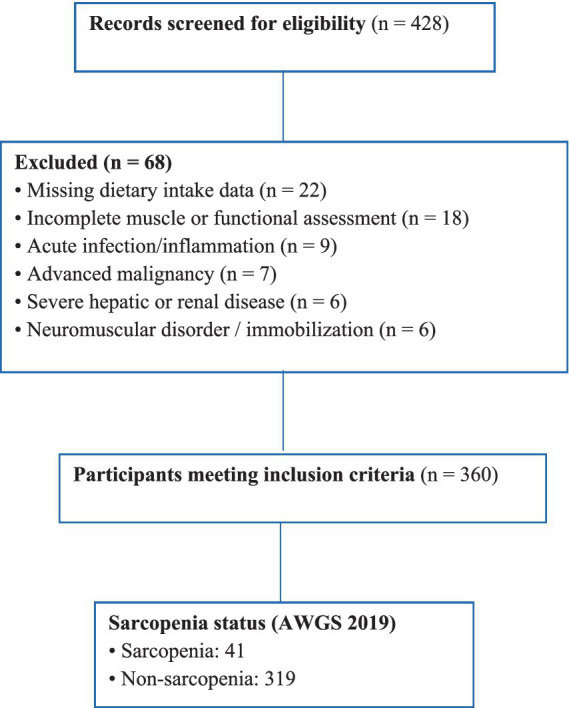
Flow diagram of participant selection.

This study was approved by the Ethics Committee of The Affiliated Huaian First People’s Hospital of Nanjing Medical University, approval number: KY-2024-240-01. This retrospective analysis was carried out utilizing data collected from routine geriatric health assessments conducted between January 2021 and December 2023. Individuals aged 60 years or older were considered for inclusion if complete information was available on anthropometric measurements, muscle mass, muscle strength, physical performance, and dietary consumption. Exclusion criteria involved acute inflammatory or infectious states at the time of evaluation advanced malignancy, serious hepatic or renal dysfunction, neuromuscular conditions, prolonged immobilization, or extended usage of medications known to significantly influence muscle metabolism, such as systemic corticosteroids. The process of participant selection is shown in Figure 1.

### 2.2 Assessment of sarcopenia

Sarcopenia was identified based on the Asian Working Group for Sarcopenia 2019 (AWGS 2019) consensus criteria. As per AWGS 2019, sarcopenia is characterized by having low appendicular skeletal muscle mass together with low muscle strength and/or poor physical performance.

#### 2.2.1 Assessment of muscle mass

Appendicular skeletal muscle mass (ASM) was determined via multi-frequency bioelectrical impedance analysis (InBody 770; InBody Co., Ltd., Seoul, Republic of Korea). It is a multi-frequency segmental bioelectrical impedance analyzer that measures body composition and computes appendicular lean mass via its built-in impedance algorithms. The device was calibrated daily following manufacturer quality-control procedures. All measurements were carried out in a temperature-controlled room (22–24 °C) by qualified clinical personnel during regular geriatric evaluations. Individuals were assessed in the morning after an overnight fast of at least 8 h. They were advised to avoid vigorous physical activity and alcohol for 24 h before evaluation and to empty their bladder right before measurement to reduce hydration-associated variability. Measurements were carried out with individuals standing barefoot on the analyzer platform, lightly holding the hand electrodes with arms bent about 15° from the trunk, following the manufacturer’s standardized operating protocol. The measurement was repeated if there was any movement or inappropriate contact during testing. Appendicular skeletal muscle mass index (ASMI) was computed as ASM (kg) divided by height squared (m^2^).

Low muscle mass was classified utilizing AWGS 2019 gender-specific threshold values:

- Men: ASMI < 7.0 kg/m^2^
- Women: ASMI < 5.7 kg/m^2^

#### 2.2.2 Assessment of muscle strength

Muscle strength was assessed by maximal handgrip strength via a calibrated digital hand dynamometer (Jamar Plus+; Patterson Medical, Warrenville, IL, USA). The device was calibrated using uniform weights once a month. Grip span was modified on an individual basis. Examination were carried out with individuals seated, shoulder adducted and neutrally rotated, elbow flexed at 90°, forearm in neutral position, and wrist positioned between 0° and 30° dorsiflexion, in accordance with standardized testing protocols. Grip strength was assessed in each hand, beginning with the dominant hand. Individuals were advised to squeeze the dynamometer with maximal effort for 3–5 s. Two trials were conducted for each hand with a rest interval of minimum 60 s between attempts. The highest value obtained from either hand was utilized for analysis.

Low muscle strength was categorized as:

- Men: handgrip strength < 28 kg
- Women: handgrip strength < 18 kg

#### 2.2.3 Assessment of physical performance

Physical performance was evaluated utilizing usual gait speed over a 6-m walking distance. Individuals were directed to walk at their habitual pace on a flat, unobstructed surface. Timing was captured via a digital stopwatch (Casio HS-80TW-1EF; Casio Computer Co., Tokyo, Japan) from the beginning of movement to the end of the 6-m course. Two trials were carried, and the faster time was utilized to compute gait speed (m/s).

Low physical performance was classified based on the AWGS 2019 as:

- Gait speed < 1.0 m/s (both sexes)

#### 2.2.4 Diagnostic criteria

As per AWGS 2019, sarcopenia was identified when low muscle mass (ASMI below the gender-specific value) coexisted with low muscle strength and/or low physical performance.

### 2.3 Anthropometric, sociodemographic, and clinical variables

Body weight and height were determined utilizing standardized methods during routine evaluations, and body mass index (BMI) was computed as kg/m^2^. Sociodemographic factors, such as age, sex, and educational attainment, were retrieved from health records. Individuals who completed primary school or less (≤6 years of formal schooling) were classified as having low education, while those who completed more than 6 years were classified as having higher education (3, 4). Physical activity was assessed via the International Physical Activity Questionnaire–Short Form (IPAQ-SF), which examines walking and moderate- and vigorous-intensity activities performed throughout the preceding 7 days. Total activity was shown in MET-min/week utilizing the standard IPAQ scoring protocol (3.3, 4.0, and 8.0 METs allocated to walking, moderate, and vigorous activities, respectively). Individuals were classified as having low physical activity (<600 MET-min/week) or adequate physical activity (≥600 MET-min/week) as per standard IPAQ criteria (Supplementary File S1). Hypertension was identified based on recorded physician diagnosis or usage of antihypertensive medicines.

### 2.4 Dietary intake assessment

Dietary consumption was evaluated utilizing three non-consecutive 24-h dietary recalls (two weekdays and one weekend day) performed by qualified registered dietitians during regular geriatric examination. Individuals documented all foods and drinks taken in the previous 24 h. Standardized multiple-pass interviewing was used to increase completeness and prevent exclusion of frequently underreported items (such as cooking oils, condiments, snacks, and drinks). Food models, verified photographic portion guides, and standardized household measuring tools were used to estimate portion sizes. When needed, caregivers aided individuals to improve reporting accuracy.

The three non-consecutive 24-h dietary recall approach utilized in this investigation is a commonly recognized and validated method for evaluating regular dietary consumption in epidemiological studies, involving older individuals. Repeated 24-h recalls have shown adequate relative reliability when contrasted with weighed dietary records and food frequency questionnaires for determining energy and macronutrient consumption. The usage of three recall days decreases within-person variations and enhances estimation of regular intake (12).

Dietary information was converted to nutrient consumption via the latest edition of the Chinese Food Composition Tables. For each individual, average daily consumption of total energy (kcal/day), protein (g/day), and dietary fiber (g/day) was computed across the three recall days to minimize within-individual day-to-day variations. Furthermore, protein consumption was expressed relative to body weight (g/kg/day).

A number of quality-control measures were used to handle possible reporting bias and implausible values. The physiologic plausibility of the total daily consumption of energy was assessed using widely recognized cut-offs in nutritional epidemiology. Intakes of less than 800 or more than 4,000 kcal per day for men and less than 600 or more than 3,500 kcal per day for women were deemed unrealistic and were not included in the analysis. Internal consistency checks were also carried out to find missing meals, incomplete recalls, or inconsistencies between disclosed portion sizes and the total amount of energy consumed. Significantly inconsistent records were excluded. The established multiple-pass recall approach, help of qualified dietitians, portion-size tools, and caregiver support (when necessary) were utilized to minimize underreporting. Random reporting error and daily variability were additionally reduced by averaging three non-consecutive recalls.

### 2.5 Statistical analysis

All statistical analyses were carried out via IBM SPSS Statistics (version 26). Continuous factors were presented as mean ± standard deviation, and categorical factors as frequencies and percentages. Differences between sarcopenic and non-sarcopenic individuals were evaluated via independent-sample t-tests or chi-square tests, as necessary. Logistic regression models were conducted to assess the relation between BMI, dietary intake factors, and sarcopenia. Three analyses were carried out: an unadjusted model (Model 1), a model adjusted for age and sex (Model 2), and a fully adjusted model further controlling for education, physical activity, and high blood pressure (Model 3). Odds ratios (ORs) with 95% confidence intervals (CIs) were reported. Dietary fiber and protein consumption were measured as absolute daily intakes (g/day). Formal energy-adjustment techniques including the residual or nutrient-density approach were not used; total energy intake was assessed as an individual exposure factor in regression tests. Subgroup analyses were also conducted by stratifying individuals based on energy consumption levels (≤1,800, 1,801–2,100, and >2,100 kcal/day), age groups (<70 and ≥70 years), and BMI categories (<23 and ≥23 kg/m^2^) (Supplementary Table S1). Multicollinearity among independent variables was evaluated utilizing variance inflation factors (VIF) obtained from linear regression models incorporating BMI, energy intake, and protein intake as predictors. A VIF value less than 2.5 was deemed indicative of absence of problematic collinearity. All tests were two-sided and *p*-value less than 0.05 was considered statistically significant.

## 3 Results

### 3.1 Participant characteristics

Out of 360 older individuals included in the analysis, 41 (11.4%) satisfied the diagnostic requirement for sarcopenia. Individuals with sarcopenia were, on average, older than those without sarcopenia (*p* = 0.001). On average, sarcopenic persons were older than non-sarcopenic individuals (*p* = 0.001). There were significant differences in educational attainment and regular physical activity between the groups; persons with sarcopenia were considerably more likely to have low levels of both education and physical activity (both *p* < 0.001). The prevalence of hypertension and gender distribution, on the other hand, were similar across groups and did not reach statistical significance (*p* = 0.194 and *p* = 0.554, respectively), indicating that sarcopenia in this cohort was more closely associated with aging and lifestyle variables than with sex or cardiometabolic comorbidity. (Table 1).

**Table 1:** Baseline characteristics of the study participants.

Variable	Non-sarcopenia (*n* = 319)	Sarcopenia (*n* = 41)	*p*-value
Age (years)	71.14 ± 5.74	74.24 ± 5.53	0.001
Male, *n* (%)	151 (47.33)	15 (36.58)	0.194
Low education, *n* (%)	102 (31.97)	24 (58.53)	<0.001
Low physical activity, *n* (%)	88 (27.58)	24 (58.53)	<0.001
Hypertension, *n* (%)	140 (43.88)	16 (39.02)	0.554
BMI (kg/m^2^)	24.70 ± 3.05	21.15 ± 2.45	<0.001
ASMI (kg/m^2^)	7.50 ± 0.55	6.45 ± 0.56	<0.001
Handgrip strength (kg)	29.00 ± 4.54	20.44 ± 4.66	<0.001
Gait speed (m/s)	1.09 ± 0.13	0.78 ± 0.13	<0.001

### 3.2 Anthropometry, muscle mass, and physical performance

Anthropometric and functional measurements showed notable variations. Sarcopenic participants’ BMI was substantially lower than that of non-sarcopenic participants (*p* < 0.001). In line with diagnosis, the sarcopenia group had a significantly lower appendicular skeletal muscle mass index (*p* < 0.001). Measures of physical performance and muscle strength corroborated these results, showing that sarcopenic individuals had considerably lower handgrip strength and slower gait speed (both *p* < 0.001). All of these findings point to a consistent deterioration in muscle quantity, strength, and functional performance in sarcopenia patients (Table 1).

### 3.3 Dietary intake

There were noticeable variations in the groups’ regular dietary intake. Compared to their non-sarcopenic counterparts, individuals with sarcopenia indicated considerably reduced total calorie intake (*p* < 0.001). Additionally, there was a significant decrease in protein intake, both in absolute terms and in relation to body weight (*p* < 0.001 for both). Additionally, sarcopenic people consumed much less dietary fiber (*p* < 0.001). These results imply that rather than a deficiency in a specific nutrient, sarcopenia was linked to a general pattern of inadequate dietary consumption (Table 2).

**Table 2:** Dietary intake according to sarcopenia status.

Variable	Non-sarcopenia	Sarcopenia	*p*-value
Energy intake (kcal/day), mean ± SD	2,104 ± 272.1	1,753 ± 287.9	<0.001
Protein intake (g/day), mean ± SD	82.35 ± 13.54	61.28 ± 11.89	<0.001
Protein intake (g/kg/day), mean ± SD	1.11 ± 0.11	0.81 ± 0.10	<0.001
Fiber intake (g/day), mean ± SD	27.29 ± 5.36	17.81 ± 4.98	<0.001

### 3.4 Association between body mass index and sarcopenia

BMI consistently and strongly demonstrated a negative correlation with sarcopenia in logistic regression analysis. A lowered BMI was significantly associated with high risks of sarcopenia in the unadjusted model (OR = 0.66, *p* < 0.001). After controlling for age and sex, this association held steady (OR = 0.65, *p* < 0.001), and it continued even after controlling for education, physical activity, and hypertension (adjusted OR = 0.64, *p* < 0.001). These findings show an inverse association between BMI and sarcopenia after controlling for demographic and lifestyle-associated variables (Table 3).

**Table 3:** BMI and sarcopenia.

Variable	Model 1 OR (95% CI)	*p*-value	Model 2 OR (95% CI)	*p*-value	Model 3 OR (95% CI)	*p*-value
BMI	0.66 (0.58–0.75)	<0.001	0.65 (0.57–0.75)	<0.001	0.64 (0.55–0.74)	<0.001

Model 1: Unadjusted.

Model 2: Adjusted for age and sex.

Model 3: Further adjusted for education, physical activity, and hypertension.

### 3.5 Association between dietary intake and sarcopenia

Increased energy consumption was substantially associated with decreased odds of sarcopenia in all regression analysis. In the unadjusted model, energy consumption (per 100 kcal/day) was negatively associated with sarcopenia (OR = 0.63, 95% CI 0.55–0.73; *p* < 0.001). This relationship remained significant after controlling for age, sex and BMI (OR = 0.65, 95% CI 0.55–0.77; *p* < 0.001) and after further correction for BMI, education, physical activity, and hypertension (OR = 0.67, 95% CI 0.56–0.80; *p* < 0.001) (Table 4).

**Table 4:** Dietary intake and sarcopenia.

Variable	Model 1 OR (95% CI)	*p*-value	Model 2 OR	*p*-value	Model 3 OR	*p*-value
Energy intake (per 100 kcal/day)	0.63 (0.55–0.73)	<0.001	0.65 (0.55–0.77)	<0.001	0.67 (0.56–0.80)	<0.001
Protein intake	0.88 (0.85–0.91)	<0.001	0.87 (0.83–0.91)	<0.001	0.85 (0.81–0.90)	<0.001
Fiber intake	0.68 (0.61–0.75)	<0.001	0.69 (0.61–0.77)	<0.001	0.70 (0.62–0.79)	<0.001

Model 1: Unadjusted.

Model 2: Adjusted for age, sex and BMI.

Model 3: Further adjusted for education, physical activity, and hypertension.

Increased protein consumption showed a significant inverse association with sarcopenia. Greater protein intake was associated with lower odds of sarcopenia in the unadjusted analysis (OR = 0.88, *p* < 0.001), and this relationship became clearer when age, sex, BMI, education, physical activity, and hypertension were taken into account (adjusted OR = 0.85, *p* < 0.001). Similarly, there was an inverse association between dietary fiber intake and sarcopenia; in both unadjusted and fully adjusted analyses, increased intake was associated with considerably lower risks (adjusted OR = 0.70, *p* < 0.001).

When considered collectively, these findings show that sarcopenia in older persons is closely associated with lower BMI and inadequate nutritional intake, especially in terms of energy, protein, and fiber. The significance of maintaining appropriate body weight and diet quality in aging populations is highlighted by the durability of these correlations following multivariable correction, which underscores the independent impact of nutritional status to sarcopenia.

Before regression analysis, multicollinearity among independent factors was evaluated. All variance inflation factor (VIF) values were less than 2.5, reflecting no sign of problematic collinearity (Table 5).

**Table 5:** Multicollinearity diagnostics.

Variable	VIF
BMI	<2.5
Energy (per 100 kcal/day)	<2.5
Protein (g/day)	<2.5

## 4 Discussion

The current retrospective study evaluated the associations among body mass index (BMI), dietary consumption, and sarcopenia in older individuals. Adults categorized as sarcopenic had substantially decreased BMI, appendicular skeletal muscle mass index, handgrip strength, gait speed, and habitual consumption of energy, protein, and dietary fiber compared with non-sarcopenic individuals. Multivariable models showed that decreased BMI and lower dietary consumption were independently associated with sarcopenia after controlling for demographic and lifestyle factors. These results indicate cross-sectional relationships within this group and do not prove causality or temporal direction.

### 4.1 Body mass index and sarcopenia

In each analytical model, lower BMI was consistently associated with higher risk of sarcopenia. This result lines up with several population-based investigations showing that lower skeletal muscle mass and diminished physical function are linked to low BMI in older persons. According to a study, the likelihood of sarcopenia in older persons was significantly correlated with decreased BMI (13). According to a further investigation, those suffering from sarcopenia had a higher 6-month mortality rate and a considerably lower BMI (14).

But because BMI is an anthropometric metric that is based on height and total body weight and does not distinguish between lean and fat mass, it is not a reliable indicator of skeletal muscle mass or body composition. Crucially, sarcopenic obesity, which is defined by the coexistence of excess adiposity and low muscle mass, is one example of a body composition phenotype that BMI fails to capture. Adults with sarcopenic obesity may have normal or higher BMI despite having less skeletal muscle mass, which could result in misclassification when BMI is utilized as a stand-in for nutritional or muscular status. Therefore, using BMI alone may underestimate sarcopenia in people who keep normal or increased body weight but have a poor body composition (15).

Furthermore, the relationship between sarcopenia and lower BMI should be interpreted cautiously. Rather than being a sign of sarcopenia, a lower BMI could be a reflection of underlying muscle loss. A gradual loss of muscle mass can help people lose weight overall, which will lower their BMI. Due to the cross-sectional nature of this investigation, it is impossible to ascertain whether a reduced BMI precedes the onset of sarcopenia or occurs as a result of it, and reverse causality cannot be eliminated.

### 4.2 Energy intake and sarcopenia

All regression analyses revealed an inverse relationship between sarcopenia and total calorie intake, which was considerably lower among sarcopenic subjects. Each 100 kcal/day rise in energy consumption was associated with lower risk of sarcopenia. Drop of body weight and lean mass in older people has long been linked to insufficient calorie intake (16, 17). Skeletal muscle tissue may be mobilized to fulfill metabolic needs when energy intake is inadequate, which can lead to gradual muscle loss. Sarcopenic people routinely consume less energy than their contemporaries who are not sarcopenic, according to observational research. According to systematic reviews and meta-analyses, older persons with sarcopenia had worse overall nutritional status and consumed less calories (8, 18). In other regions, comparable correlations have been discovered among older persons.

The current results are in line with earlier observational studies that found relationships between increased total energy intake and improved muscle-associated variables in later life. The efficiency of dietary protein intake may also be limited by insufficient energy since maintaining muscle protein requires adequate energy.

### 4.3 Protein intake and sarcopenia

Sarcopenia was found to be strongly inversely correlated with protein consumption. In comparison to non-sarcopenic individuals, sarcopenic individuals ingested substantially lower protein both in absolute terms and per kilogram of body weight. These results are in line with a large body of research showing a strong correlation between dietary protein intake and functional outcomes, muscle mass, and strength in older persons (19, 20). Previous research has associated increased protein intake to better lean body mass trajectories in older adults (21, 22). Sarcopenia is less common in those who consume more protein on a regular basis, according to systematic reviews and meta-analyses (5).

Importantly, compared to younger people, older adults have anabolic resistance and need to consume more protein in order to enhance muscle protein synthesis (MPS). Research suggests that maintaining muscle strength and function in older persons requires daily intakes of 1.0–1.2 g/kg body weight, which is greater than the conventional 0.8 g/kg RDA (23). Reduced protein consumption has been associated with a greater prevalence of sarcopenia-associated factors, as evidenced by another community screening that determined older people with protein intake ≤1 g/kg/day were more likely to have lower lean mass, reduced grip strength, and impaired physical performance, particularly among women (24). In the current study, protein ingestion among sarcopenic persons was below levels frequently related with favorable muscle outcomes, supporting the finding that decreased protein consumption is a nutritional trait frequently seen in people with sarcopenia.

### 4.4 Dietary fiber and sarcopenia

Individuals with sarcopenia had considerably reduced dietary fiber consumption, which was independently associated with sarcopenia even after controlling for lifestyle and demographic factors. Increased dietary fiber intake was independently linked to improved physical performance metrics in older individuals, such as faster gait speed, longer walking distance, and stronger handgrips, according to a large community-centered investigation (25). In the NU-AGE cohort of older persons, skeletal muscle mass index was considerably greater in those who consumed more fiber than in those who did not (26).

A recently conducted cross-sectional investigation utilizing data from the Korea National Health and Nutrition Examination Survey discovered that older women in the lowest quartile of fiber consumption were more likely to have low muscle strength than those in the highest quartile among individuals aged ≥65 years (11). Another study found that the sarcopenia-prone elderly group should consume a sufficient amount of dietary fiber (10). When taken as a whole, these studies consistently show links between decreased dietary fiber consumption and sarcopenia or sarcopenia-associated factors in older individuals.

### 4.5 Clinical implications

The results of this study support the clinical significance of nutritional status in connection to older persons’ sarcopenia. Regularly gathered clinical and nutritional data may be useful in identifying people with compromised muscle health, as evidenced by the constant correlations found between lower body mass index (BMI), decreased dietary consumption, and sarcopenia. BMI is a straightforward anthropometric metric that is frequently used in medical settings. Regular BMI monitoring may help identify elderly individuals who might benefit from additional functional status and muscle strength assessments as part of a thorough geriatric evaluation. The observed relationships between sarcopenia and decreased energy and protein consumption highlight the significance of recoding habitual dietary consumption in older persons. In therapeutic practice, basic nutritional evaluation may offer further context when assessing older adults with poor BMI, decreased strength, or functional constraints. When combined, these results demonstrate the value of using basic nutritional indicators, such as BMI and dietary intake measurements, in addition to evaluations of older persons’ physical performance and muscular strength. Clinicians may find this information useful in identifying people who have poorer muscle health and dietary vulnerability.

### 4.6 Strengths

There are various strengths of this study:

- First, a set of international consensus standards that included objective measurements of muscle mass, muscle strength, and physical performance were used to define sarcopenia. Relative to studies that rely on a single sign, the incorporation of many components for sarcopenia diagnosis increases the validity of case categorization and lowers the possibility of misclassification.
- Second, the investigation looked at body mass index and a number of dietary consumption factors at the same time, such as total energy, protein, and dietary fiber intake. A broader overview of nutritional status in connection to sarcopenia was provided by this thorough nutritional examination, which enabled assessment of overall dietary sufficiency rather than concentrating on a single nutrient.
- Third, using multivariable regression methods, the analysis took into consideration a number of significant confounding variables, such as age, sex, education level, physical activity, and hypertension. The consistency of relationships after correction strengthens confidence that the identified links were not solely explained by these factors.
- Lastly, the inclusion of older persons 60 years of age and older increases the findings’ applicability to older individuals frequently seen in clinical and public health settings.

### 4.7 Limitations

It is important to take into account the limitations of this study when evaluating the results:

- First, the cross-sectional and retrospective design makes it impossible to draw conclusions about causality. The noticed associations between reduced BMI, decreased dietary consumption, and sarcopenia may reflect reverse causation. It is conceivable that sarcopenia, via decreased physical ability or appetite, may led to reduced body weight and lower nutritional intake. Longitudinal investigations are necessary to elucidate temporal links.
- Second, nutritional consumption was measured using self-reported 24-h recalls, which are prone to reporting errors and recollection bias, especially in older persons.
- Third, despite the fact that the models were corrected for age, sex, education, physical activity, and hypertension, a number of significant confounding variables were left out. Lower muscle mass and worse physical performance in older individuals have been linked to diabetes mellitus (27). Likewise, muscle loss and functional decline have been associated with chronic kidney disease (28). Also, lower muscle mass and strength in older populations have been linked to elevated inflammatory markers (29). Furthermore, a thorough evaluation of medication use was lacking. Systemic corticosteroids, some antidiabetic medications, diuretics, and statins are among the frequently prescribed medications for older adults that may affect body weight, appetite, muscle mass, or function (30).
- Finally, the research cohort was drawn from a single dataset, which may restrict generalizability to other groups with different demographic, cultural, or dietary features.

## 5 Conclusion

The currents findings demonstrated a clear association between nutritional status and sarcopenia in older persons. Subjects with lower body mass index and less than ideal regular dietary intake showed poorer muscle mass, strength, and physical performance. These findings highlight how crucial it is to continue consuming enough energy in addition to enough protein and dietary fiber as people age. From a clinical standpoint, identifying older persons at higher risk of sarcopenia may be aided by routine assessment of body weight and dietary habits. Integrating nutritional evaluation into geriatric care may offer additional context for tailored dietary counseling, although prospective investigations are needed to ascertain whether such strategies alter sarcopenia trajectories.

## Data Availability

The raw data supporting the conclusions of this article will be made available by the authors without undue reservation.
