# Supplementary material for: Body mass index and dietary intake as nutritional determinants of sarcopenia in older adults
Source: Front Nutr. 2026 May 4;13:1798406. doi: 10.3389/fnut.2026.1798406 (PMC13181808; doi:10.3389/fnut.2026.1798406)
Supplement: Supplementary file 1 [file Table_1.DOCX]

Supplementary Table S1: Subgroup analysis of sarcopenia prevalence according to BMI categories, age groups, and energy intake levels among older adults (n = 360)

| **Variable** | **Category** | **N** | **Sarcopenia (n)** | **Prevalence (%)** | **p-value** |
| --- | --- | --- | --- | --- | --- |
| **BMI category (kg/m²)** | <23 | 120 | 30 | 25.0 | <0.001 |
|  | ≥23 | 240 | 11 | 4.6 |  |
| **Age group (years)** | <70 | 133 | 7 | 5.3 | 0.005 |
|  | ≥70 | 227 | 34 | 15.0 |  |
| **Energy Intake Category (kcal/day)** | ≤1800 | 69 | 23 | 33.3 | <0.001 |
|  | 1801–2100 | 117 | 13 | 11.1 |  |
|  | >2100 | 174 | 5 | 2.9 |  |
