# Supplementary material for: Body mass index and dietary intake as nutritional determinants of sarcopenia in older adults
Source: Front Nutr. 2026 May 4;13:1798406. doi: 10.3389/fnut.2026.1798406 (PMC13181808; doi:10.3389/fnut.2026.1798406)
Supplement: Supplementary file 2 [file Table_2.DOCX]

**International Physical Activity Questionnaire – Short Form (IPAQ-SF)**

**Instructions for Participants**

The following questions ask about the time you spent being physically active during the last 7 days. Please answer each question even if you do not consider yourself an active person.

Physical activity includes activities done at work, during transportation, household chores, exercise, and recreational activities.

Please report only activities performed for at least 10 minutes at a time.

**Section A – Vigorous Physical Activity**

Vigorous activities refer to activities that require substantial physical effort and cause rapid breathing or a significant increase in heart rate.

Examples include:

- Running or jogging
- Fast cycling
- Aerobic exercise
- Competitive sports
- Heavy manual labor
- Carrying heavy loads

**Question A1**

During the last 7 days, on how many days did you perform vigorous physical activities for at least 10 minutes at a time?

Number of days: ______ days/week (0–7)

**Question A2**

On the days when you performed vigorous activities, how much time did you usually spend doing these activities per day?

Hours: ______
Minutes: ______

**Section B – Moderate Physical Activity**

Moderate activities refer to activities that require moderate physical effort and cause a noticeable but moderate increase in breathing or heart rate.

Examples include:

- Brisk walking
- Light cycling
- Carrying light loads
- Household chores (mopping, sweeping)
- Gardening
- Recreational activities such as badminton or doubles tennis

**Question B1**

During the last 7 days, on how many days did you perform moderate physical activities for at least 10 minutes at a time?

Number of days: ______ days/week (0–7)

**Question B2**

On those days, how much time did you usually spend doing moderate physical activities per day?

Hours: ______
Minutes: ______

**Section C – Walking**

Walking includes walking performed for transportation, exercise, or daily activities, such as:

- Walking to work or the market
- Walking for recreation or exercise
- Walking inside the home or neighborhood

**Question C1**

During the last 7 days, on how many days did you walk for at least 10 minutes at a time?

Number of days: ______ days/week (0–7)

**Question C2**

On those days, how much time did you usually spend walking per day?

Hours: ______
Minutes: ______

**Section D – Sedentary Behaviour (Sitting Time)**

Sedentary behaviour provides additional context for physical activity patterns.

**Question D1**

During the last 7 days, how much time did you usually spend sitting on a typical weekday?

Include time spent sitting while:

- Watching television
- Reading
- Using a computer or smartphone
- Sitting during transportation
- Sitting at work or home

Hours: ______
Minutes: ______

**Data Processing and Scoring**

Responses were converted to **MET-minutes per week** using the standardized IPAQ scoring protocol.

Assigned metabolic equivalent (MET) values:

| **Activity Type** | **MET Value** |
| --- | --- |
| Walking | 3.3 METs |
| Moderate activity | 4.0 METs |
| Vigorous activity | 8.0 METs |

Total physical activity was calculated as:

$$MET\text{-}min/week=(Walking\times3.3)+(Moderate\times4.0)+(Vigorous\times8.0)$$

**Physical Activity Classification**

Participants were categorized according to established IPAQ criteria:

| **Category** | **Definition** |
| --- | --- |
| Low physical activity | <600 MET-min/week |
| Adequate physical activity | ≥600 MET-min/week |
